# Perception and willingness toward various immunization routes for COVID-19 vaccines: a cross-sectional survey in China

**DOI:** 10.3389/fpubh.2023.1192709

**Published:** 2023-09-25

**Authors:** Haohang Wang, Mingting Cui, Shunran Li, Fan Wu, Shiqiang Jiang, Hongbiao Chen, Jianhui Yuan, Caijun Sun

**Affiliations:** ^1^School of Public Health (Shenzhen), Shenzhen Campus of Sun Yat-sen University, Shenzhen, China; ^2^Nanshan District Center for Disease Control and Prevention, Shenzhen, China; ^3^Department of Epidemiology and Infectious Disease Control, Longhua Key Discipline of Public Health for the Prevention and Control of Infectious Diseases, Longhua Centre for Disease Control and Prevention, Shenzhen, China; ^4^School of Public Health (Shenzhen), Sun Yat-sen University, Guangzhou, China; ^5^Key Laboratory of Tropical Disease Control (Sun Yat-sen University), Ministry of Education, Guangzhou, China

**Keywords:** COVID-19 vaccine, immunization route, booster, needle fear, vaccine hesitancy

## Abstract

**Background:**

To date, most vaccines, including the COVID-19 vaccine, are mainly administered by intramuscular injection, which might lead to vaccine hesitancy in some populations due to needle fear. Alternatively, needle-free immunization technology is extensively developed to improve the efficacy and acceptance of vaccination. However, there is no study to report the perception and willingness toward various immunization routes of the COVID-19 vaccine in the general population.

**Methods:**

A cross-sectional survey was conducted nationwide using an online questionnaire. Bivariate analyses were undertaken to assess variable associations among the participants who reported a hesitancy to receive the COVID-19 booster vaccination. Multivariable logistic regression with a backward step-wise approach was used to analyze the predicted factors associated with the willingness to receive the COVID-19 booster vaccination.

**Results:**

A total of 3,244 valid respondents were included in this survey, and 63.2% of participants thought they had a good understanding of intramuscular injection, but only 20.7, 9.2, 9.4, and 6.0% of participants had a self-perceived good understanding of inhalation vaccine, nasal spray vaccine, oral vaccine, and microneedle patch vaccine. Correspondingly, there was high acceptance for intramuscular injection (76.5%), followed by oral inhalation (64.4%) and nasal spray (43.0%). Those participants who were only willing to receive an intramuscular vaccine had less vaccine knowledge (OR = 0.78; 95% CI: 0.65–0.94) than those who were willing to receive a needle-free vaccine (OR = 1.97; 95% CI: 1.52–2.57). Some factors were found to be associated with vaccine hesitancy toward booster COVID-19 vaccination.

**Conclusion:**

Needle-free vaccination is a promising technology for the next generation of vaccines, but we found that intramuscular injection was still the most acceptable immunization route in this survey. One major reason might be that most people lack knowledge about needle-free vaccination. We should strengthen the publicity of needle-free vaccination technology, and thus improve the acceptance and coverage of vaccination in different populations.

## 1. Introduction

The coronavirus disease 2019 (COVID-19) pandemic caused by the severe acute respiratory syndrome coronavirus 2 (SARS-CoV-2) is still a serious challenge for global public health (1). To eventually control this pandemic, mass vaccination of the general population is extensively considered the most cost-effective intervention. As of 1 March 2023, more than 13.32 billion doses of COVID-19 vaccines had been used worldwide, of which the top three countries in terms of cumulative doses were China (3.49 billion), India (2.21 billion), and the United States (672 million) (2).

A variety of COVID-19 vaccines, including inactivated vaccines, protein subunit-based vaccines, mRNA-based vaccines, and recombinant viral vector-based vaccines, have been well-developed over the last 3 years. These vaccines are safe and effective in the early stages of the pandemic against SARS-CoV-2 infections and their related clinical symptoms (3, 4). However, the effectiveness of existing COVID-19 vaccines has waned due to the emerging SARS-CoV-2 variants (5, 6), especially for the omicron variants and their sub-lineages, including BA.5.2, BF.7, BQ.1.1, XBB.1.5, and CH.1.1 (7, 8). To promote the efficacy of the next generation of COVID-19 vaccines, various strategies are being extensively developed, including novel antigen design, prime/boost immunization, and induction of mucosal immunity with needle-free vaccination (oral inhalation, nasal spray, and oral capsule) (9–11). In addition, as a result of the government's effective control of the pandemic, there has been a decrease in people's acceptance of the COVID-19 vaccine when compared with the initial stages of the COVID-19 outbreak. Therefore, it is of great importance to enhance the public's willingness to vaccinate for the long-term prevention of the COVID-19 pandemic (12).

Currently, at least 15 kinds of COVID-19 vaccines have been approved by the WHO for human use, and most of these vaccines are administered via the intramuscular route. However, there are some limitations to intramuscular injection-based vaccination, including a relatively weak mucosal immunity against SARS-CoV-2, medical personnel, and cold-chain equipment requirements. More importantly, some people might have needle fear or needle phobia due to the trauma and pain of intramuscular injection, which may cause vaccine hesitancy (13, 14). Needle-free technology has the potential to increase the willingness and availability of vaccinations by eliminating the fear and discomfort associated with needles.

However, the public may not appropriately have access to these advantages and thus have limited knowledge or misunderstandings about needle-free vaccines, which can hinder people's acceptance of vaccination, particularly when faced with novel vaccine (15). A previous study has shown that if a vaccine is recommended by the government or officials, it can effectively increase the public's willingness to be vaccinated. Therefore, disseminating precise and timely information about novel vaccine technology by a highly trusted government might contribute to improving vaccination coverage (16). To date, there is no study to report the knowledge and willingness toward various immunization routes of COVID-19 vaccines among the general population in China. Therefore, we conducted this study to address this issue in Chinese citizens, and we also investigated how people choose the various immunization routes of COVID-19 vaccines and the potential factors that contribute to vaccine hesitancy for multiple boosters of COVID-19 vaccination.

## 2. Methods

### 2.1. Study design

We conducted a cross-sectional study using an anonymous online questionnaire from 34 provincial administrative regions in China between 16 November 2022 and 5 December 2022. During this period, booster doses of COVID-19 vaccines were available to the adult population that had received the basic two doses of COVID-19 vaccines over 6 months. The secondary booster dose of COVID-19 was also recommended for the susceptible population.

### 2.2. Participants' recruitment

Individuals were recruited as participants if they were: (1) able to read and complete the online self-administered questionnaire independently; (2) informed consent to participate in the study. In addition, participants who had incorrect quality control question answers were excluded. The participants' recruitment relied on convenience sampling by disseminating the study questionnaire's URL link or quick response (QR) code on WeChat (https://weixin.qq.com/), which is currently regarded as the most popular instant messaging platform in China.

The sample size was calculated using PASS 21.0.3 (https://www.ncss.com/). A sample size of 789 was produced by a two-sided 95% confidence interval (CI) with a width equal to 0.04, and the proportion of participants with a hesitancy to receive a COVID-19 booster vaccine was estimated as 0.084 according to a recent study (17–19). The formula is as follows:


$$\begin{array}{l} N=\left[\frac{Z_{1-\frac{\alpha }{2}}^{2}}{{\left(\frac{\varepsilon }{2}\right)}^{2}}\right]\times p\times \left(1-p\right) \end{array}$$

*N* is the sample size. *Z* is the Z-score corresponding to the desired level of confidence. For a two-sided 95% CI, the Z-score is ~1.96. α = 0.05. *p* is the estimated proportion of participants with a characteristic of interest. ε is the width of CI equal to 0.04. When *p* is unknown, we can set *p* = 0.5, and *N* is equal to 2,401.

### 2.3. Measurement and data collection

The questionnaire used in this study consists of four structures (Supplementary material): (1) Basic demographic information and health condition; (2) pandemic experiences and vaccination status; (3) participants' knowledge and attitudes regarding different immunization routes of COVID-19 vaccination; and (4) the modified Vaccine Hesitancy Scale (VHS).

### 2.4. Cognition on vaccine knowledge

Considering that limited knowledge or misunderstanding about needle-free vaccination might be a barrier for people to access needle-free vaccination (20), we therefore investigated the cognition of these participants toward self-perceived advantages and disadvantages between intramuscular injection and needle-free vaccination. As we know, healthy adult populations who have completed regular immunization are recommended to vaccinate with the orally aerosolized Ad5-nCoV (Convidecia Air), which is the first needle-free COVID-19 vaccine approved for emergency use by the China government in September 2022. Based on recent knowledge (21, 22), incorrect options were intentionally set in our questionnaire with reverse scoring, which are: (1) advantages: “Needle-free vaccines usually last a longer immune response” and “Needle-free vaccines usually have less side effects” (Actually, there were comparable immune duration and side effects between orally aerosolized Ad5-nCoV vaccine and intramuscularly injected Ad5-nCoV vaccine); and (2) disadvantages: “The time for needle-free vaccines to induce immune response is more slower” and “Needle-free vaccines are inconvenient to administer” (Actually, compared with the traditional intramuscularly injected vaccine, the time for this vaccine to induce an immune response is similar, and the administration of needle-free vaccines is more convenient). Participants who self-reported unawareness of needle-free vaccines or got a zero scores were identified as the incorrect understanding group, and the other participants were identified as the correct understanding group.

### 2.5. Vaccine hesitancy

In this study, we used the Vaccine Hesitancy Scale (VHS), which was developed by the WHO Strategic Advisory Group on Experts (SAGE) Working Group (23), to quantify participants' hesitancy to COVID-19 booster vaccination. The terms in the original VHS were modified by our experienced researchers in order to fit the latest Chinese COVID-19 epidemic situation and vaccine development. The modified scale had 10 items, and each item was asked on a 5-point Likert scale (strongly disagree, disagree, neither agree nor disagree, agree, and strongly agree). Hesitancy to receive a COVID-19 booster vaccine was defined as individuals with 30 scores or less of a total of 50 scores using the hesitancy scale. Exploratory factor analysis (EFA) and confirmatory factor analysis (CFA) indicated that this scale had good reliability and validity (Supplementary Tables 2, 3), consistent with our previous studies (24–26).

### 2.6. Statistical analysis

Descriptive analyses were conducted to describe the involved participants. Frequencies and proportions were calculated for categorical variables, and continuous variables were summarized by mean and standard deviation. Bivariate analyses were then undertaken to assess variable associations in the group of respondents who reported a hesitancy to receive a COVID-19 booster vaccine. To determine which factors might be associated with this hesitancy, we then conducted a multivariate logistic regression to estimate odds ratios (ORs) with 95% confidence intervals (CIs) with a backward step-wise approach. Factors with a *P* < 0.2 in the bivariate analyses for hesitancy were included in the logistic regression model. Data were analyzed using R 4.1.3 and SPSS 26.0 (IBM). Statistical significance was set at a *P* < 0.05.

### 2.7. Ethics approval and consent to participate

The protocol for this study was approved by the Ethics Committee of the School of Public Health (Shenzhen), Sun Yat-sen University (approval number: SYSU-PHS-IACUC-2022-065). Participation was voluntary, and informed consent was obtained at the beginning of the questionnaire.

## 3. Result

### 3.1. Participants' characteristics and their status of vaccine hesitancy

A total of 3,244 (82.9%) of the 3,911 respondents recruited in this survey were considered valid. Participants' mean (±SD) age was 31.81 (±8.88) years, and 91.6% of them were aged between 18 and 44 years old. For the educational level, 66% of the participants had reached a bachelor's degree or even a higher degree. In addition, 151 (4.7%) participants suffered from various chronic diseases (Table 1).

**Table 1:** Descriptive characteristics of populations by hesitancy to receive COVID-19 vaccine booster doses in China.

**Variables**	**Total [*n* (%)]**	**Vaccine hesitancy**		***p***
		**Hesitancy [*****n*** **(%)]**	**Non-hesitancy [*****n*** **(%)]**	
Sample size	3,244 (100)	376 (11.6)	2,868 (88.4)	
Age, year [mean (SD)]	31.81 (8.88)	31.92 (8.82)	31.79 (8.89)	0.789
**Sex**				
Male	754 (23.2)	109 (29.0)	645 (22.5)	0.006^*^
Female	2,490 (76.8)	267 (71.0)	2,223 (77.5)	
**Marriage and bearing**				
Single	594 (18.3)	77 (20.5)	517 (18.0)	0.278
Married and without children	51 (1.6)	6 (1.6)	45 (1.6)	
Married and keeping children	2,579 (79.5)	293 (77.9)	2,286 (79.7)	
Others	20 (0.6)	0 (0.0)	20 (0.7)	
**Educational level**				
≤ Junior high school	411 (12.7)	43 (11.4)	368 (12.8)	0.001^*^
Senior high school	694 (21.4)	74 (19.7)	620 (21.6)	
Bachelor	1,974 (60.9)	224 (59.6)	1,750 (61.0)	
≥Master	165 (5.1)	35 (9.3)	130 (4.5)	
**Occupation**				
Businessman	238 (7.3)	32 (8.5)	206 (7.2)	0.016^*^
Farmer	46 (1.4)	5 (1.3)	41 (1.4)	
Healthcare worker	109 (3.4)	10 (2.7)	99 (3.5)	
Company employee or professional technician	954 (29.4)	127 (33.8)	827 (28.8)	
Public servant	202 (6.2)	25 (6.6)	177 (6.2)	
Student	526 (16.2)	68 (18.1)	458 (16.0)	
Teacher	146 (4.5)	12 (3.2)	134 (4.7)	
Unemployment or housework	65 (2.0)	4 (1.1)	61 (2.1)	
Ordinary worker/farm laborer	200 (6.2)	31 (8.2)	169 (5.9)	
Others	758 (23.4)	62 (16.5)	696 (24.3)	
**Area**				
Urban	3,054 (94.1)	349 (92.8)	2,705 (94.3)	0.296
Rural	190 (5.9)	27 (7.2)	163 (5.7)	
**Monthly income (RMB)**				
≤ 5,000	1,337 (41.2)	146 (38.8)	1,191 (41.5)	0.006^*^
5,001–10,000	1,201 (37.0)	138 (36.7)	1,063 (37.1)	
10,001–15,000	404 (12.5)	39 (10.4)	365 (12.7)	
≥15,001	302 (9.3)	53 (14.1)	249 (8.7)	
**Chronic diseases**				
No	3,093 (95.3)	353 (93.9)	2,740 (95.5)	0.193
Yes	151 (4.7)	23 (6.1)	128 (4.5)	

* *p* < 0.05.

Consistent with the recommended vaccination procedures at the time of our investigation, 3,059 (94.3%) of participants had received the regular two doses of COVID-19 vaccines, and 2,314 (71.3%) of participants had received another booster dose of COVID-19 vaccines.

According to the score of the VHS and its classified criteria, 2,868 (88.4%) of participants were positive for COVID-19 vaccination, while 376 (11.6%) of participants were verified to be in a state of vaccine hesitancy. The further chi-square test showed that participants of different sexes, educational levels, occupations, and monthly incomes had different levels of vaccine hesitancy.

### 3.2. Knowledge and acceptance of various immunization routes of COVID-19 vaccines

**Figure 1:**
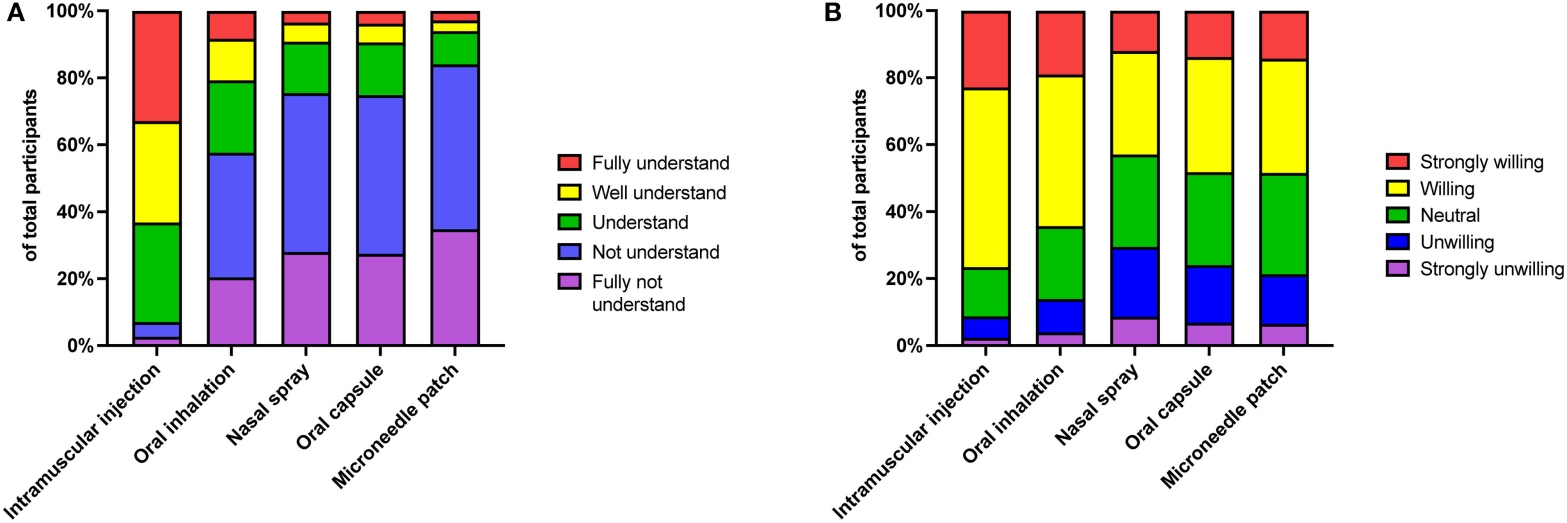
Knowledge and acceptance of various immunization routes of COVID-19 vaccines. Distribution of the understanding levels **(A)** and acceptance degree **(B)** toward various immunization routes of COVID-19 vaccines, including intramuscular injection, oral inhalation, nasal spray, oral capsule, and microneedle patch.

Among different immunization routes of COVID-19 vaccines, 63.2% of participants thought they had a good understanding (fully understood or well-understood) of the immunization route of intramuscular injection. By contrast, there were only 20.7, 9.2, 9.4, and 6.0% of participants had a self-perceived good understanding of inhalation vaccine, nasal spray vaccine, oral vaccine, and microneedle patch vaccine, respectively (Figure 1A). Consistent with the level of understanding toward various immunization routes of vaccination, there was the strongest acceptance for intramuscular injection (76.5%), followed by oral inhalation (64.4%), nasal spray (43.0%), oral capsule (48.2%), and microneedle patch (48.4%; Figure 1B).

**Figure 2:**
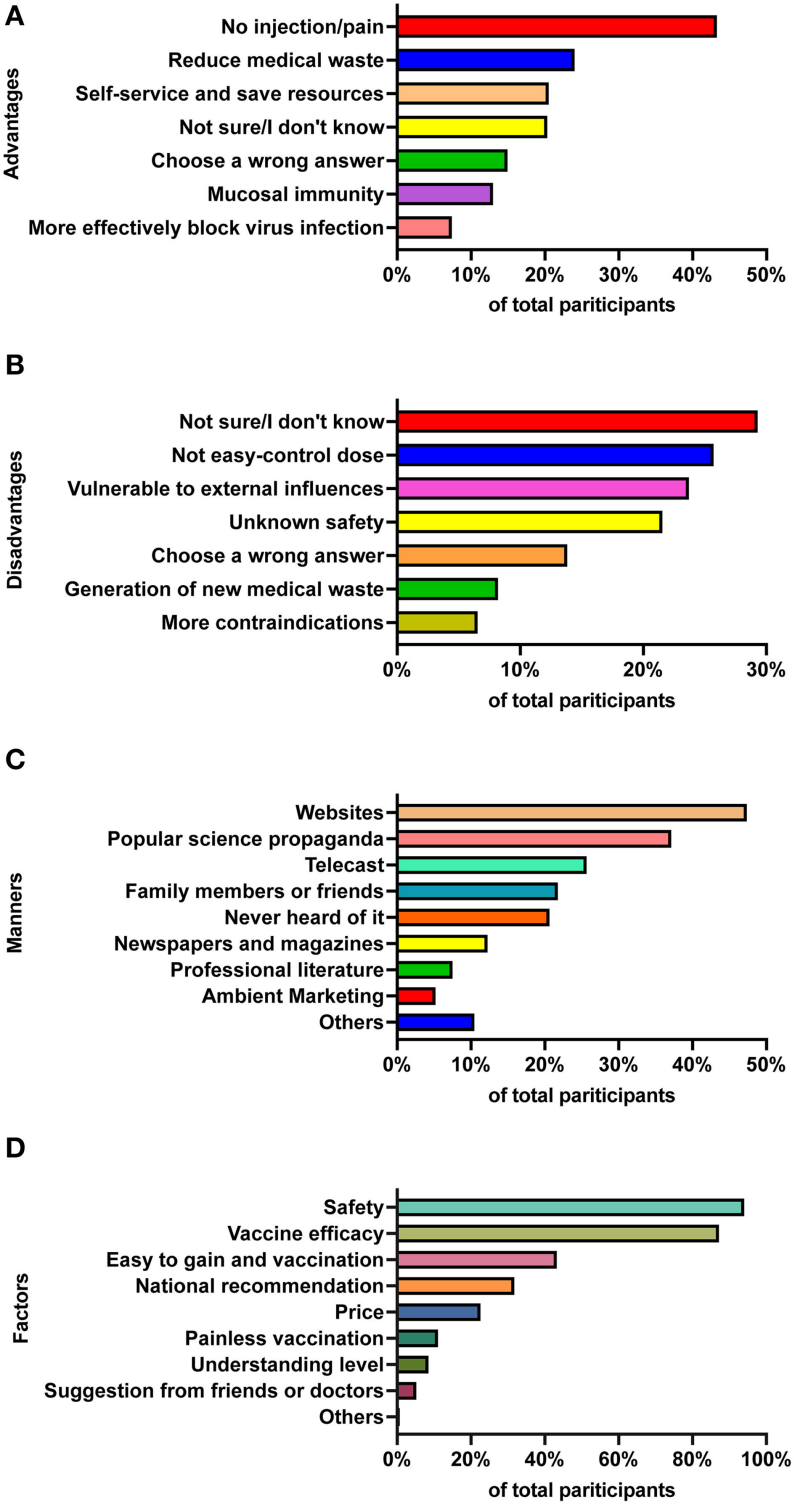
Participants' perception and knowledge about needle-free immunization and the associated factors. Participants' perception of advantages **(A)** and disadvantages **(B)** between traditional intramuscular injected vaccines and novel needle-free vaccines. **(C)** Manners of participants to access information about various COVID-19 vaccines. **(D)** Main factors associated with participants' willingness to get vaccination.

We then investigated the participants' cognition about the advantages and disadvantages of traditional intramuscular injection vaccines and burgeoning needle-free vaccines. The majority (43.3%) thought that the main advantage of needle-free immunization was “no injection/no pain,” 24.0% thought that the main advantage was “reduce medical waste,” and 20.5% thought that needle-free immunization could be “self-service and save medical resources.” By contrast, only 13.0% of participants chose the induction of mucosal immunity, and 7.4% chose “More effectively block virus infection.” On the other side, the top three options for disadvantages for participants were “Not sure/I don't know” (29.3%), “Not easy to control the doses” (25.7%), and “Vulnerable to external influences (such as cough and sneeze)” (23.7%). Of note, as for these incorrect options, ~15% of participants chose at least one incorrect answer for each question, implying that these participants had limited knowledge and misunderstandings about needle-free immunization (Figures 2A, B).

Considering that needle-free vaccines are likely to be the most promising immunization routine in future, we specifically investigated people's knowledge about needle-free immunization and the associated factors. In our survey, we found that 47.3% of participants acquired vaccine knowledge from internet websites, 37.0% from popular science propaganda at workplaces or schools, and 25.0% from telecasts (Figure 2C). Of note, 20.6% of participants chose “never heard it,” indicating that this population did care about vaccine-related information. In addition, most participants regarded safety (94.0%) and vaccine efficacy (87.1%) as the main factors when receiving a vaccination, followed by “easy to gain and vaccination” (43.2%), “National recommendations” (31.7%), and “price” (22.5%; Figure 2D).

Further analysis demonstrated that there was a statistical correlation between the participants' vaccine knowledge level and the ways to access vaccine information. Those who obtained vaccine information through websites, newspapers, magazines, telecasts, and professional literature had a higher score than those who obtained it in other unofficial ways or never cared about the vaccine information (Table 2 and Supplementary Table 4). Interestingly, those participants who were only willing to receive an intramuscular vaccine had a lower cognition score (incorrect understanding) than those who were willing to receive a needle-free vaccine. As expected, participants with a higher education level had a higher frequency of acquiring the correct vaccine information.

**Table 2:** Multivariate logistic regression of vaccine knowledge level and information acquisition about intramuscular injection vaccine and needle-free vaccine.

**Variables**	**Incorrect understanding**	**Correct understanding**	**Multivariate**	***p***
	**(*****n*** = **1,644, %)**	**(*****n*** = **1,600, %)**	**aOR**^#^ **(95%CI)**	
**Age, year [mean (SD)]**	32.65 (8.34)	30.94 (9.33)	0.99 [0.98, 1.00]	0.114
**Sex**				
Male	341 (20.7)	413 (25.8)	1 (ref.)	
Female	1,303 (79.3)	1,187 (74.2)	0.84 [0.69, 1.03]	0.095
**Education level**				
≤ Junior high school	258 (15.7)	153 (9.6)	1 (ref.)	
Senior high school	383 (23.3)	311 (19.4)	1.40 [1.05, 1.86]	0.022^*^
Bachelor	952 (57.9)	1,022 (63.9)	1.60 [1.23, 2.09]	0.001^*^
≥Master	51 (3.1)	114 (7.1)	2.55 [1.57, 4.18]	< 0.001^*^
**Occupation**				
Company employee or professional technician	553 (33.6)	401 (25.1)	1 (ref.)	
Student	160 (9.7)	366 (22.9)	2.20 [1.61, 3.01]	< 0.001^*^
Businessman	121 (7.4)	117 (7.3)	1.27 [0.92, 1.76]	0.150
Public servant	116 (7.1)	86 (5.4)	1.09 [0.77, 1.56]	0.628
Ordinary worker/farm laborer	101 (6.1)	99 (6.2)	1.70 [1.19, 2.44]	0.004^*^
Teacher	59 (3.6)	87 (5.4)	1.90 [1.28, 2.85]	0.002^*^
Healthcare worker	22 (1.3)	87 (5.4)	4.51 [2.62, 8.06]	< 0.001^*^
Unemployment or housework	36 (2.2)	29 (1.8)	1.33 [0.74, 2.39]	0.345
Farmer	35 (2.1)	11 (0.7)	0.52 [0.24, 1.07]	0.088
Others	441 (26.8)	317 (19.8)	1.14 [0.91, 1.43]	0.256
**Way to access information about needle-free vaccines for COVID-19**				
**Websites**				
No	1,067 (64.9)	641 (40.1)	1 (ref.)	
Yes	577 (35.1)	959 (59.9)	1.25 [1.05, 1.50]	0.013^*^
**Newspapers and magazines**				
No	1,527 (92.9)	1,320 (82.5)	1 (ref.)	
Yes	117 (7.1)	280 (17.5)	1.56 [1.22, 2.01]	< 0.001^*^
**Telecast**				
No	1,358 (82.6)	1,054 (65.9)	1 (ref.)	
Yes	286 (17.4)	546 (34.1)	1.47 [1.23, 1.78]	< 0.001^*^
**Professional literature**				
No	1,580 (96.1)	1,421 (88.8)	1 (ref.)	
Yes	64 (3.9)	179 (11.2)	1.57 [1.14, 2.18]	0.007^*^
**Never heard of it**				
No	1,045 (63.6)	1,530 (95.6)	1 (ref.)	
Yes	599 (36.4)	70 (4.4)	0.12 [0.09, 0.16]	< 0.001^*^
**Others**				
No	1,464 (89.1)	1,441 (90.1)	1 (ref.)	
Yes	180 (10.9)	159 (9.9)	0.73 [0.57, 0.95]	0.018^*^
**Willingness on another booster vaccination against COVID-19**				
All vaccination methods are acceptable	680 (41.4)	647 (40.4)	1 (ref.)	
Intramuscular injection	603 (36.7)	473 (29.6)	0.78 [0.65, 0.94]	0.008^*^
Needle-free vaccine	137 (8.3)	307 (19.2)	1.97 [1.52, 2.57]	< 0.001^*^
Other routes of vaccination	68 (4.1)	91 (5.7)	1.23 [0.85, 1.80]	0.274
Unwilling to be vaccinated again	130 (7.9)	61 (3.8)	0.58 [0.40, 0.84]	0.004^*^
Not vaccinated yet	26 (1.6)	21 (1.3)	1.06 [0.54, 2.07]	0.866
**Frequency of attention to news reports about COVID-19 vaccines**				
≥Once a day	473 (28.8)	509 (31.8)	1.18 [0.97, 1.44]	0.093
≥Once a week	503 (30.6)	639 (39.9)	0.69 [0.56, 0.85]	0.001^*^
Community education or message prompt	573 (34.9)	416 (26.0)	0.34 [0.21, 0.55]	< 0.001^*^
Never care	95 (5.8)	36 (2.2)	1.18 [0.97, 1.44]	0.093

* p < 0.05;

# aOR, adjusted odd ratio; ref, reference. Bivariate analysis is presented in Supplementary Table 4.

### 3.3. Factors associated with vaccine hesitancy toward booster COVID-19 vaccination

Previous studies showed that needle phobia (“Afraid of needles”) might be a factor associated with vaccine hesitancy (27–29), and we thus investigated this topic in this study. In our questionnaire, we defined those participants as having “Potential needle-phobia” if they were: (1) not vaccinated due to fear of needles or (2) regarded the pain as the main factor to affect whether receiving vaccination. However, Pearson's chi-square test analysis showed that participants who were considered “potential needle-phobia” had no statistical difference between other participants on the status of vaccine hesitancy, indicating that injection fear may not be a major factor that led to vaccine hesitancy in the Chinese population (Supplementary Table 1).

We further found that these people who had gotten at least one dose of the COVID-19 vaccine, willing to receive an intramuscular injection/inhalation or oral vaccine, were more likely to accept the COVID-19 booster vaccination. Participants who chose “not sure or didn't know about the advantages of needle-free vaccine” were less likely to accept the COVID-19 booster vaccination (Table 3). In addition, these people who had a high education level (graduate student or above) and a high income (15,000 RMB and above/per month) were also less likely to accept the COVID-19 booster vaccination. However, sex, occupation, age, residence place, or chronic disease condition were not significantly correlated with vaccine hesitancy toward COVID-19 booster vaccination (Supplementary Table 5).

**Table 3:** Multivariate logistic regression of factors associated with vaccine hesitancy toward booster COVID-19 vaccination.

**Variables**	**Non-hesitancy**	**Hesitancy**	**Multivariate**	***p***
	**(*****n*** = **2,868, 100%)**	**(*****n*** = **376, 100%)**	**aOR**^#^ **(95%CI)**	
**Sex**				
Male	645 (22.5)	109 (29.0)	1 (ref.)	
Female	2,223 (77.5)	267 (71.0)	0.73 [0.57, 0.95]	0.017^*^
**Marriage and bearing**				
Single	517 (18.0)	77 (20.5)	1 (ref.)	
Married and without children	45 (1.6)	6 (1.6)	0.64 [0.23, 1.54]	0.358
Married and keeping children	2,286 (79.7)	293 (77.9)	0.80 [0.56, 1.14]	0.212
Others	20 (0.7)	0 (0.0)	0.00 [NA, 97.24]	0.965
**Educational level**				
≤ Junior high school	368 (12.8)	43 (11.4)	1 (ref.)	
Senior high school	620 (21.6)	74 (19.7)	1.07 [0.71, 1.63]	0.744
Bachelor	1,750 (61.0)	224 (59.6)	1.12 [0.78, 1.64]	0.555
≥Master	130 (4.5)	35 (9.3)	2.36 [1.35, 4.09]	0.002^*^
**Area**				
Urban	2,705 (94.3)	349 (92.8)	1 (ref.)	
Rural	163 (5.7)	27 (7.2)	1.43 [0.89, 2.25]	0.130
**Monthly income (RMB)**				
≤ 5,000	1,191 (41.5)	146 (38.8)	1 (ref.)	
5,001–10,000	1,063 (37.1)	138 (36.7)	1.16 [0.87, 1.55]	0.311
10,001–15,000	365 (12.7)	39 (10.4)	0.94 [0.61, 1.42]	0.769
≥15,001	249 (8.7)	53 (14.1)	1.74 [1.16, 2.58]	0.006^*^
**Vaccination procedure**				
Unvaccinated	64 (2.2)	23 (6.1)	1 (ref.)	
Partial	87 (3.0)	11 (2.9)	0.32 [0.14, 0.71]	0.006^*^
Regular	645 (22.5)	100 (26.6)	0.44 [0.26, 0.77]	0.003^*^
One dose booster	2,029 (70.7)	235 (62.5)	0.30 [0.18, 0.51]	< 0.001^*^
Two doses or more booster	43 (1.5)	7 (1.9)	0.42 [0.15, 1.08]	0.085^*^
**Willingness of various COVID-19 vaccines**				
**Intramuscular injection**				
No	614 (21.4)	147 (39.1)	1 (ref.)	
Yes	2,254 (78.6)	229 (60.9)	0.56 [0.44, 0.72]	< 0.001^*^
**Oral capsule**				
No	962 (33.5)	194 (51.6)	1 (ref.)	
Yes	1,906 (66.5)	182 (48.4)	0.71 [0.52, 0.95]	0.021^*^
**Oral inhalation**				
No	1,428 (49.8)	252 (67.0)	1 (ref.)	
Yes	1,440 (50.2)	124 (33.0)	0.67 [0.49, 0.91]	0.011^*^
**Know about advantages of needle-free vaccine**				
No	1,504 (52.4)	234 (62.2)	1 (ref.)	
Yes	1,364 (47.6)	142 (37.8)	0.75 [0.59, 0.96]	0.022^*^

* *p* < 0.05;

# aOR, adjusted odd ratio; ref, reference. Bivariate analysis is presented in Supplementary Table 5.

## 4. Discussion

Previous studies suggested that the needle fear due to intramuscular injection might play a role in vaccine hesitancy in some countries. For example, a survey conducted among 15,014 UK adults revealed that 3,927 (26.2%) were positive for blood-injection-injury phobia (14). A meta-analysis including 35 studies also yielded similar results, estimating that the prevalence of needle fear ranged from 20 to 50% in adolescents and 20–30% in young adults (13). However, in the present study, there were only 11.1% of participants identified “potential needle phobia,” and we also found that needle fear might not be the primary factor leading to vaccine hesitancy in the Chinese population, since there was no statistical correlation between the potential needle-phobia participants and their status of vaccine hesitancy. One potential reason for this difference might be the Chinese government's and medical personnel's vigorous promotion of the COVID-19 vaccines since the start of the COVID-19 epidemic. As a result, Chinese people are willing to believe that COVID-19 vaccination is beneficial to protect themselves and control the pandemic, resulting in high COVID-19 vaccination coverage and effective control of the COVID-19 pandemic (30, 31). The second reason for this observation might be that most people have insufficient knowledge and cognition about various immunization routes, especially for these novel needle-free vaccination technologies, including inhalation vaccine, nasal spray vaccine, oral vaccine, and microneedle patch vaccine. Consequently, intramuscular injection is still the most acceptable immunization route in this survey, mainly because most participants thought they had a better understanding of intramuscular injection than that of other needle-free vaccines. Thus, it is necessary to promote public cognition about the advantages and disadvantages of intramuscular injection-based vaccines and needle-free vaccines.

Recent studies have demonstrated that these needle-free vaccines are more efficacious against pathogen infection when compared with traditional intramuscular vaccines (32–34). However, there were only a few participants who chose the advantage of needle-free immunization as “induction of mucosal immunity” (13.0%), and “more effectively block virus infection (7.4%).” Instead, many participants in our investigation thought that the main advantages of needle-free immunization were “no injection/no pain” (43.3%), “reduce medical waste (24.0%),” and “self-service and save medical resources (20.5%).” Thus, approximately half of the participants did not understand well or had limited knowledge and misunderstandings about the efficacy of needle-free vaccines (Figures 1, 2). This is interesting to note because “induction of mucosal immunity” and “more effectively block virus infection” might not be easily understood by the general public, but the explanations of “no injection/no pain” and “reduce medical waste” can be easily understood. Therefore, we should optimize some options in future research to make them easier to understand for the public.

The mindsponge theory, an emerging theory to illustrate how the human mind receives and filters information, and accepts or rejects values (35), may provide a framework to explain our above results. According to this theory, the emerging values are compared with the existing values in an individual's core mindset by a multi-filtering system, and then the advantages and disadvantages of accepting or rejecting the emerging values are assessed. Therefore, people's cognition of intramuscular injection-based vaccines and trust in the government can be thought of as existing values in an individual's core mindset, which will affect the evaluation and acceptance of emerging information regarding needle-free vaccines. In our study, ~20% of participants expressed concern regarding the safety of needle-free vaccines. Considering that most participants regarded safety (94.0%) and vaccine efficacy (87.1%) as the main factors when receiving vaccination, the limited knowledge and misunderstanding about needle-free immunization might be the main factor to hinder the implementation of the needle-free COVID-19 vaccines. Previous survey showed that Chinese people had a high proportion (83.7%) of positive responses when they were asked if they would accept a vaccine recommended by the government (36), which was consistent with our result in this study (87.3%, 2,835 of 3,244). According to the mindsponge theory, people will be inclined to accept emerging information provided by an existing value of a highly trusted government. Thus, we also emphasize the importance for people to obtain vaccine information from official channels instead of other ways, since inaccurate information from unreliable sources may affect people's cognition of COVID-19 vaccines (20, 37). As a result, the government and authoritative social media should strengthen the publicity of needle-free vaccination technology, and thus improve the acceptance and coverage of vaccination in different populations.

Our study has limitations. First, this is a cross-sectional study, and we could not conclude the causal relationship between vaccine hesitancy and knowledge of various immunization routes. Therefore, more similar studies are needed to further clarify how people's perceptions of different routes of vaccination affect vaccination intentions. Second, our questionnaire's participants relied on convenience sampling through social media, which has inherent recruitment biases. As a result, it may be challenging to generalize the findings to different populations. However, this limitation could be partially addressed by using directed invitations to specific population groups. To obtain a more precise assessment of the population's perception and willingness, additional studies using more representative sampling methods are needed. Therefore, while our study provides valuable insights, these results should be interpreted with caution.

## 5. Conclusion

Overall, needle fear may not be the primary factor causing vaccine hesitancy in the Chinese population, and intramuscular injection remains the most acceptable immunization route in this survey, mainly because most people lack knowledge about needle-free vaccination. Additionally, it is of great importance for the public to obtain correct information about novel vaccination technology from reliable sources. We should strengthen the publicity of needle-free vaccination technology, and thus improve the acceptance and coverage of vaccination in different populations.

## Data availability statement

The raw data supporting the conclusions of this article will be made available by the authors, without undue reservation.

## Ethics statement

The studies involving humans were approved by Ethics Committee of the School of Public Health (Shenzhen), Sun Yat-sen University (Approval number: SYSU-PHS-IACUC-2022-065). The studies were conducted in accordance with the local legislation and institutional requirements. The participants provided their written informed consent to participate in this study.

## Author contributions

CS conceived and designed this project and revised and edited the manuscript. HW, MC, SL, and FW performed this project, analyzed the data, and drafted the manuscript. SJ, HC, and JY contributed resources and discussion. All authors have read and agreed to the published version of the manuscript.
