# Supplementary material for: Perception and willingness toward various immunization routes for COVID-19 vaccines: a cross-sectional survey in China
Source: Front Public Health. 2023 Sep 25;11:1192709. doi: 10.3389/fpubh.2023.1192709 (PMC10560725; doi:10.3389/fpubh.2023.1192709)
Supplement: Supplementary file 1 [file Data_Sheet_1.DOCX]

**Supplementary materials**

**Supplementary Table 1. Pearson's Chi-squared test with Yates' continuity correction of potential needle-phobia and vaccine hesitancy.**

|  | hesitancy | non-hesitancy | χ^2^ | df | *p* |
| --- | --- | --- | --- | --- | --- |
| potential needle-phobia | 343 | 2541 | 2.0634 | 1 | 0.1509 |
| non-potential needle-phobia | 33 | 327 |  |  |  |

**Supplementary Table 2. EFA loadings and CFA standardized regression weights of the 10-item scale**

| Vaccine hesitancy scale items | EFA Component matrix after rotation | | |  | CFA Standardized regression weights (n = 3244) | | | |
| --- | --- | --- | --- | --- | --- | --- | --- | --- |
|  | Confidence | Complacency | Cost |  | Confidence | Complacency | | Cost |
| A COVID-19 vaccine booster can protect my health | 0.626 | -0.175 | -0.090 |  | 0.569 |  |  | |
| I think a COVID-19 vaccine booster will be very effective in preventing COVID-19 | 0.801 | -0.077 | -0.041 |  | 0.716 |  |  | |
| A COVID-19 vaccine can protect people (family, friends, colleagues) around me from infection | 0.779 | -0.017 | -0.046 |  | 0.673 |  |  | |
| If there is booster for the new SARS-COV-2 strain, I will try to get it immediately | 0.796 | -0.128 | -0.099 |  | 0.767 |  |  | |
| If the COVID-19 vaccine booster is recommended by the government, doctors, the community and other professionals, I believe vaccination is beneficial | 0.828 | -0.164 | -0.058 |  | 0.848 |  |  | |
| I will recommend people around me (family, friends, colleagues) to take every dose of COVID-19 vaccine | 0.832 | -0.173 | -0.095 |  | 0.863 |  |  | |
| I have already received enough doses of COVID-19 vaccine, so there‘s no need to receive the booster | -0.150 | 0.874 | 0.183 |  |  | 0.798 |  | |
| The COVID-19 pandemic has been alleviated, and there is no need to be vaccinated against COVID-19 | -0.198 | 0.874 | 0.148 |  |  | 0.821 |  | |
| I am still worrying about the possible side effects of a COVID-19 vaccine | -0.074 | 0.100 | 0.827 |  |  |  | 0.537 | |
| If the COVID-19 vaccine booster is paid partially or completely by my own expense, I may not choose to get vaccinated | -0.097 | 0.187 | 0.766 |  |  |  | 0.608 | |

**Note: 120 samples were randomly selected for exploratory factor analysis (EFA). EFA = Exploratory Factor Analysis. CFA = Confirmatory Factor Analysis. Extraction Method: Principal Axis Factoring (PAF); Rotation Method: Varimax with Kaiser Normalization.**

**Supplementary Table 3. Comparison of the construct validity by CFA**

| . | $\chi^{2}/df$ | SRMR | RMSEA | CFI | NNFI-TLI |
| --- | --- | --- | --- | --- | --- |
| 3 factors | 20.640 | 0.032 | 0.078 | 0.952 | 0.933 |
| 2 factors | 25.089 | 0.0434 | 0.086 | 0.938 | 0.918 |
| Suggested value for good fit | 2~3 | ＜0.05 | ＜0.06 | ＞0.90 | ＞0.90 |

**Note: A CFA (n=3244) was conducted for the Comparison between 2-3 factor composition. As shown, the model fitting indexes of 3 factors are better than those of 2 factors.**

**Supplementary Table 4. Bivariate logistic regression of vaccine knowledge level and information acquisition about intramuscular injection vaccine and needle-free vaccine.**

| **Variables** | **Incorrect understanding** | **Correct understanding** | **Bivariate** | ***p*** |
| --- | --- | --- | --- | --- |
|  | **(n=1644, %)** | **(n=1600, %)** | **OR (95%CI)** |  |
| **Age, year [mean (SD)]** | 32.65 (8.34) | 30.94 (9.33) | 0.98 [0.97, 0.99] | <0.001* |
| **Sex** |  |  |  |  |
| Male | 341 (20.7) | 413 (25.8) | 1 (ref.) |  |
| Female | 1303 (79.3) | 1187 (74.2) | 0.75 [0.64, 0.89] | 0.001* |
| **Marriage and bearing** |  |  |  |  |
| Single | 190 (11.6) | 404 (25.2) | 1 (ref.) |  |
| Married and without children | 27 (1.6) | 24 (1.5) | 0.42 [0.23, 0.74] | 0.003* |
| Married and keeping children | 1417 (86.2) | 1162 (72.6) | 0.39 [0.32, 0.47] | <0.001* |
| Others | 10 (0.6) | 10 (0.6) | 0.47 [0.19, 1.16] | 0.098 |
| **Education level** |  |  |  |  |
| ≤Junior high school | 258 (15.7) | 153 (9.6) | 1 (ref.) |  |
| Senior high school | 383 (23.3) | 311 (19.4) | 1.37 [1.07, 1.76] | 0.014* |
| Bachelor | 952 (57.9) | 1022 (63.9) | 1.81 [1.46, 2.26] | <0.001* |
| ≥ Master | 51 (3.1) | 114 (7.1) | 3.77 [2.58, 5.58] | <0.001* |
| **Occupation** |  |  |  |  |
| Company employee or professional technician | 553 (33.6) | 401 (25.1) | 1 (ref.) |  |
| Student | 160 (9.7) | 366 (22.9) | 3.15 [2.52, 3.96] | <0.001* |
| Businessman | 121 (7.4) | 117 (7.3) | 1.33 [1.00, 1.77] | 0.048* |
| Public servant | 116 (7.1) | 86 (5.4) | 1.02 [0.75, 1.39] | 0.888 |
| Unskilled worker/farm laborer | 101 (6.1) | 99 (6.2) | 1.35 [1.00, 1.84] | 0.053 |
| Teacher | 59 (3.6) | 87 (5.4) | 2.03 [1.43, 2.91] | <0.001* |
| Healthcare worker | 22 (1.3) | 87 (5.4) | 5.45 [3.42, 9.06] | <0.001* |
| Unemployment or housework | 36 (2.2) | 29 (1.8) | 1.11 [0.67, 1.84] | 0.684 |
| Farmer | 35 (2.1) | 11 (0.7) | 0.43 [0.21, 0.84] | 0.017* |
| Others | 441 (26.8) | 317 (19.8) | 0.99 [0.82, 1.20] | 0.929 |
| **Area** |  |  |  |  |
| Urban | 1576 (95.9) | 1478 (92.4) | 1 (ref.) |  |
| Rural | 68 (4.1) | 122 (7.6) | 1.91 [1.42, 2.61] | <0.001* |
| **Monthly income (RMB)** |  |  |  |  |
| ≤5000 | 631 (38.4) | 706 (44.1) | 1 (ref.) |  |
| 5001-10000 | 665 (40.5) | 536 (33.5) | 0.72 [0.62, 0.84] | <0.001* |
| 10001-15000 | 207 (12.6) | 197 (12.3) | 0.85 [0.68, 1.06] | 0.154 |
| ≥15001 | 141 (8.6) | 161 (10.1) | 1.02 [0.79, 1.31] | 0.873 |
| **Chronic disease** |  |  |  |  |
| No | 1577 (95.9) | 1516 (94.8) | 1 (ref.) |  |
| Yes | 67 (4.1) | 84 (5.2) | 1.30 [0.94, 1.82] | 0.113 |
| **Way to access information about needle-free vaccines of COVID-19** | | | | |
| **Websites** |  |  |  |  |
| No | 1067 (64.9) | 641 (40.1) | 1 (ref.) |  |
| Yes | 577 (35.1) | 959 (59.9) | 2.77 [2.40, 3.19] | <0.001* |
| **Newspapers and magazines** |  |  |  |  |
| No | 1527 (92.9) | 1320 (82.5) | 1 (ref.) |  |
| Yes | 117 (7.1) | 280 (17.5) | 2.77 [2.21, 3.49] | <0.001* |
| **Ambient Marketing** |  |  |  |  |
| No | 1578 (96.0) | 1498 (93.6) | 1 (ref.) |  |
| Yes | 66 (4.0) | 102 (6.4) | 1.63 [1.19, 2.25] | 0.003* |
| **Telecast** |  |  |  |  |
| No | 1358 (82.6) | 1054 (65.9) | 1 (ref.) |  |
| Yes | 286 (17.4) | 546 (34.1) | 2.46 [2.09, 2.90] | <0.001* |
| **Popular science propaganda from school or workplace** | | | | |
| No | 1176 (71.5) | 864 (54.0) | 1 (ref.) |  |
| Yes | 468 (28.5) | 736 (46.0) | 2.14 [1.85, 2.48] | <0.001* |
| **Family members or friends** |  |  |  |  |
| No | 1340 (81.5) | 1198 (74.9) | 1 (ref.) |  |
| Yes | 304 (18.5) | 402 (25.1) | 1.48 [1.25, 1.75] | <0.001* |
| **Professional literature** |  |  |  |  |
| No | 1580 (96.1) | 1421 (88.8) | 1 (ref.) |  |
| Yes | 64 (3.9) | 179 (11.2) | 3.11 [2.33, 4.20] | <0.001* |
| **Never heard of it** |  |  |  |  |
| No | 1045 (63.6) | 1530 (95.6) | 1 (ref.) |  |
| Yes | 599 (36.4) | 70 (4.4) | 0.08 [0.06, 0.10] | <0.001* |
| **Others** |  |  |  |  |
| No | 1464 (89.1) | 1441 (90.1) | 1 (ref.) |  |
| Yes | 180 (10.9) | 159 (9.9) | 0.90 [0.72, 1.12] | 0.347 |
| **Willingness on another booster vaccination against COVID-19** | | | | |
| All vaccination methods are acceptable | 680 (41.4) | 647 (40.4) | 1 (ref.) |  |
| Intramuscular injection | 603 (36.7) | 473 (29.6) | 0.82 [0.70, 0.97] | 0.019* |
| Needle-free vaccine | 137 (8.3) | 307 (19.2) | 2.36 [1.88, 2.97] | <0.001* |
| Other routes of vaccination | 68 (4.1) | 91 (5.7) | 1.41 [1.01, 1.97] | 0.044* |
| Unwilling to be vaccinated again | 130 (7.9) | 61 (3.8) | 0.49 [0.36, 0.68] | <0.001* |
| Not vaccinated yet | 26 (1.6) | 21 (1.3) | 0.85 [0.47, 1.52] | 0.583 |
| **Frequency of attention to news reports about COVID-19 vaccines** | | | | |
| ≥ Once a day | 473 (28.8) | 509 (31.8) | 1 (ref.) |  |
| ≥ Once a week | 503 (30.6) | 639 (39.9) | 1.18 [0.99, 1.40] | 0.057 |
| Community education or message prompt | 573 (34.9) | 416 (26.0) | 0.67 [0.56, 0.81] | <0.001* |
| Never care | 95 (5.8) | 36 (2.2) | 0.35 [0.23, 0.52] | <0.001* |

*, *p* < 0.05; ref, reference.

**Supplementary Table 5. Bivariate logistic regression of factors associated with vaccine hesitancy towards booster COVID-19 vaccination**

| **Variables** | **Non-hesitancy** | **Hesitancy** | **Bivariate** | ***p*** |
| --- | --- | --- | --- | --- |
|  | **(n=2868, 100%)** | **(n=376, 100%)** | **OR (95%CI)** |  |
| **Age, year [mean (SD)]** | 31.79 (8.89) | 31.92 (8.82) | 1.00 [0.99, 1.01] | 0.789 |
| **Sex** |  |  |  |  |
| Male | 645 (22.5) | 109 (29.0) | 1 (ref.) |  |
| Female | 2223 (77.5) | 267 (71.0) | 0.71 [0.56, 0.91] | 0.005* |
| **Marriage and bearing** |  |  |  |  |
| Single | 517 (18.0) | 77 (20.5) | 1 (ref.) |  |
| Married and without children | 45 (1.6) | 6 (1.6) | 0.90 [0.33, 2.02] | 0.806 |
| Married and keeping children | 2286 (79.7) | 293 (77.9) | 0.86 [0.66, 1.13] | 0.273 |
| Others | 20 (0.7) | 0 (0.0) | 0.00 [NA, 188.11] | 0.967 |
| **Educational level** |  |  |  |  |
| ≤Junior high school | 368 (12.8) | 43 (11.4) | 1 (ref.) |  |
| Senior high school | 620 (21.6) | 74 (19.7) | 1.02 [0.69, 1.53] | 0.917 |
| Bachelor | 1750 (61.0) | 224 (59.6) | 1.10 [0.78, 1.56] | 0.605 |
| ≥ Master | 130 (4.5) | 35 (9.3) | 2.30 [1.41, 3.75] | 0.001* |
| **Occupation** |  |  |  |  |
| Company employee or professional technician | 827 (28.8) | 127 (33.8) | 1 (ref.) |  |
| Student | 458 (16.0) | 68 (18.1) | 0.97 [0.70, 1.32] | 0.834 |
| Businessman | 206 (7.2) | 32 (8.5) | 1.01 [0.66, 1.52] | 0.957 |
| Public servant | 177 (6.2) | 25 (6.6) | 0.92 [0.57, 1.43] | 0.721 |
| Unskilled worker/farm laborer | 169 (5.9) | 31 (8.2) | 1.19 [0.77, 1.81] | 0.414 |
| Teacher | 134 (4.7) | 12 (3.2) | 0.58 [0.30, 1.04] | 0.088 |
| Healthcare worker | 99 (3.5) | 10 (2.7) | 0.66 [0.31, 1.24] | 0.225 |
| Unemployment or housework | 61 (2.1) | 4 (1.1) | 0.43 [0.13, 1.06] | 0.105 |
| Farmer | 41 (1.4) | 5 (1.3) | 0.79 [0.27, 1.87] | 0.633 |
| Others | 696 (24.3) | 62 (16.5) | 0.58 [0.42, 0.80] | 0.001* |
| **Area** |  |  |  |  |
| Urban | 2705 (94.3) | 349 (92.8) | 1 (ref.) |  |
| Rural | 163 (5.7) | 27 (7.2) | 1.28 [0.83, 1.93] | 0.246 |
| **Monthly income (RMB)** |  |  |  |  |
| ≤5000 | 1191 (41.5) | 146 (38.8) | 1 (ref.) |  |
| 5001-10000 | 1063 (37.1) | 138 (36.7) | 1.06 [0.83, 1.36] | 0.649 |
| 10001-15000 | 365 (12.7) | 39 (10.4) | 0.87 [0.59, 1.25] | 0.469 |
| ≥15001 | 249 (8.7) | 53 (14.1) | 1.74 [1.22, 2.43] | 0.002* |
| **Chronic diseases** |  |  |  |  |
| No | 2740 (95.5) | 353 (93.9) | 1 (ref.) |  |
| Yes | 128 (4.5) | 23 (6.1) | 1.39 [0.86, 2.16] | 0.154 |
| **Vaccination procedure** |  |  |  |  |
| Unvaccinated | 64 (2.2) | 23 (6.1) | 1 (ref.) |  |
| Partial | 87 (3.0) | 11 (2.9) | 0.35 [0.15, 0.76] | 0.009* |
| Regular | 645 (22.5) | 100 (26.6) | 0.43 [0.26, 0.74] | 0.002* |
| One dose booster | 2029 (70.7) | 235 (62.5) | 0.32 [0.20, 0.54] | <0.001* |
| Two doses or more booster | 43 (1.5) | 7 (1.9) | 0.45 [0.17, 1.10] | 0.095 |
| **Willingness of various COVID-19 vaccine** | | | | |
| **Intramuscular injection** |  |  |  |  |
| No | 614 (21.4) | 147 (39.1) | 1 (ref.) |  |
| Yes | 2254 (78.6) | 229 (60.9) | 0.42 [0.34, 0.53] | <0.001* |
| **Oral capsule** |  |  |  |  |
| No | 962 (33.5) | 194 (51.6) | 1 (ref.) |  |
| Yes | 1906 (66.5) | 182 (48.4) | 0.47 [0.38, 0.59] | <0.001* |
| **Nasal spray** |  |  |  |  |
| No | 1591 (55.5) | 259 (68.9) | 1 (ref.) |  |
| Yes | 1277 (44.5) | 117 (31.1) | 0.56 [0.45, 0.71] | <0.001* |
| **Inhalation** |  |  |  |  |
| No | 1428 (49.8) | 252 (67.0) | 1 (ref.) |  |
| Yes | 1440 (50.2) | 124 (33.0) | 0.49 [0.39, 0.61] | <0.001* |
| **Microneedle patch** |  |  |  |  |
| No | 1427 (49.8) | 246 (65.4) | 1 (ref.) |  |
| Yes | 1441 (50.2) | 130 (34.6) | 0.52 [0.42, 0.65] | <0.001* |
| **Know about advantages of needle-free vaccine** | | | | |
| No | 1504 (52.4) | 234 (62.2) | 1 (ref.) |  |
| Yes | 1364 (47.6) | 142 (37.8) | 0.67 [0.54, 0.83] | <0.001* |
| **Know about disadvantages of needle-free vaccine** |  |  |  |  |
| No | 1780 (62.1) | 255 (67.8) | 1 (ref.) |  |
| Yes | 1088 (37.9) | 121 (32.2) | 0.78 [0.62, 0.97] | 0.030* |

*, *p* < 0.05; ref, reference.

**Questionnaire: Survey of** **knowledge and willingness towards various** **immunization routes of COVID-19 vaccines**

**Dear friends:** We are a survey team from Sun Yat-sen University. The purpose of this survey is to inquire about the public's knowledge and willingness towards various immunization routes of COVID-19 vaccines. The responses of this survey will only be used for academic research and will not disclose your personal information. The survey is completed anonymously, please answer objectively as much as possibles.

**Do you agree to participate in this survey?** (if you under the age of 18, please fill with the parents' accompanying)

○ Yes

○ No

1. What is your gender? [Single choice]

○ Male

○ Female

2. Your age is_________ old [fill in the blanks]

3. What is your educational background? [Single choice]

○ Junior high school and below

○ Senior high school (including technical school and technical secondary school)

○ University or undergraduate

○ Master or above

4. Your marriage and childbearing situation is [single choice]

○ Unmarried

○ Married without children

○ Married and childbearing

○ Others_________________

5. What is your occupation? [Single choice]

○ Student

○ Businessmen

○ Service industry (including artists)

○ Teacher

○ Company Officer

○ Workers

○ Government Officer

○ Healthcare worker

○ Farmer

○ Retired

○ Others_________________

6. Your current living region is: [single choice]

○ Urban area (including villages in the city)

○ Rural/mountainous areas

7. What is your monthly income? [Single choice]

○ ≤ 5000 yuan

○ 5001-10000 yuan

○ 10001-15000 yuan

○ ≥ 15001 yuan

8. Do you suffer the following chronic diseases? [Multiple choice]

□ Tumor

□ Hypertension

□ diabetes

□ Other chronic diseases requiring long-term medication

□ None of the above

9. In the past three months, how often have you actively paid attention to news reports about COVID-19 vaccine? [Single choice]

○ Very concerned, at least once a day

○ Relatively concerned, at least once a week

○ Not very concerned. I will only see the news reports pushed by the social media or community

○ Basically, don't pay attention and don't read relevant information carefully

10. What is your current progress of COVID-19 vaccination? [Single choice]

○ No vaccination (please skip to question 14)

○ Incomplete vaccination (only one dose of inactivated vaccine or one or two doses of recombinant subunit vaccine) (please skip to question 12)

○ Completion of basic immunization (two doses of inactivated vaccine, one dose of adenovirus vector vaccine or three doses of recombinant subunit vaccine) (please skip to question 12)

○ Inoculated with enhancer times (the third dose of inactivated vaccine, the second dose of adenovirus vector vaccine or the fourth dose of recombinant subunit vaccine) (please skip to question 12)

○ Inoculated the fourth dose of vaccine (inoculated again after the above booster dose) (please skip to question 12)

11. Have you ever had the following symptoms within three days after you received any COVID-19 vaccine? [Multiple choice]

□ Pain, swelling or induration at the vaccination site

□ Fever

□ Fatigue

□ Headache

□ Muscle soreness

□ Others, please describe: _________________

□ None of the above

Rely on item 2 of question 10; 3; 4; 5 options

12. After which dose did you have such symptoms? [Multiple choice]

□ First dose

□ Second dose

□ Third dose

□ Fourth dose

□ Others_________________

Rely on item 1 of item 11; 2; 3; 4; 5; 6 options*

13. Why did you not receive the COVID-19 vaccine? [Multiple choice]

□ I have a contraindication of COVID-19 vaccination, or have other conditions that need to be postponed

□ I am very healthy and do not need to be vaccinated with COVID-19 vaccine

□ COVID-19 has been controlled, and COVID-19 vaccine does not need to be inoculated again

□ I am worried about the safety and effectiveness of COVID-19 vaccine

□ I feel scared or nervous about the pain/injection scene

□ Others_________________

Depends on the first option in question 10*

14. In the past month, the number of times you have received nucleic acid testing is [single choice]

○ ≤ 3 times

○ 4-10 times

○ 11-20 times

○ ≥ 20 times

15 Have you ever had a COVID-19 antigen test? [Single choice]

○ Yes

○ No

16. Have you or people around you (such as family members, neighbors, friends or colleagues) ever been infected with SARS-COV-2? [Single choice]

○ Yes

○ No

○ Not clear

17. Have you ever experienced isolation or control related to COVID-19? [Single choice]

○ Yes

○ No

The following is an introduction to different immunization routes of COVID-19 vaccines (pictures are search from the Internet):

1.
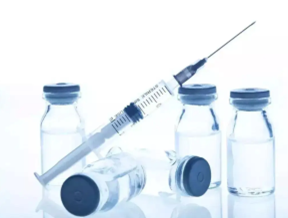
Intramuscular injection:

Inject the vaccine into the muscle tissue through a syringe to work

1.
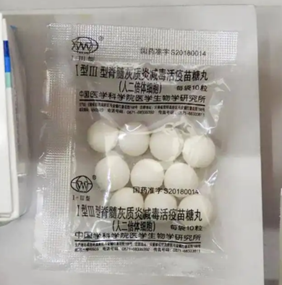
Oral capsule:


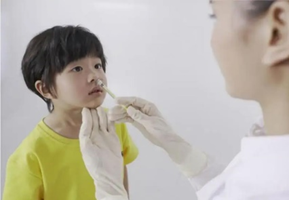
Make the vaccine into sugar pills or liquid for oral use

1. Nasal spray:

Spray the vaccine into the nasal cavity and take effect after inhalation

1.
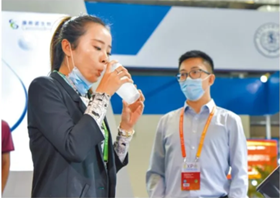
Oral inhalation:

Vaccine inoculated by inhalation after atomization

1.
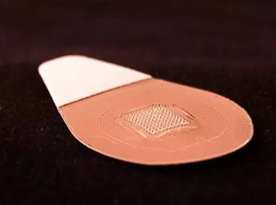
Microneedle patch:

Vaccine that can be inoculated by sticking the patch on the skin like a band-aid

18. How much do you understand about the following immunization routes [Matrix questions]

|  | Fully understand | Well understand | understand | not understand | Fully not understand |
| --- | --- | --- | --- | --- | --- |
| Intramuscular injection | ○ | ○ | ○ | ○ | ○ |
| Oral capsule | ○ | ○ | ○ | ○ | ○ |
| Nasal spray | ○ | ○ | ○ | ○ | ○ |
| Oral inhalation | ○ | ○ | ○ | ○ | ○ |
| Microneedle patch | ○ | ○ | ○ | ○ | ○ |

19. If you are sure to be vaccinated, are you willing to vaccinate in the above immunization routes? [Matrix questions]

|  | Strongly willing | Willing | Neutral | Unwilling | Strongly unwilling |
| --- | --- | --- | --- | --- | --- |
| Intramuscular injection | ○ | ○ | ○ | ○ | ○ |
| Oral capsule | ○ | ○ | ○ | ○ | ○ |
| Nasal spray | ○ | ○ | ○ | ○ | ○ |
| Oral inhalation | ○ | ○ | ○ | ○ | ○ |
| Microneedle patch | ○ | ○ | ○ | ○ | ○ |

20. How do you know the information about the “needle free” COVID-19 vaccine? [Up to three items] [multiple choice]

□ Website (including active online search)

□ Newspapers and magazines

□ Ambient Marketing

□ Telecast

□ Popular science propaganda from school or workplace

□ Family members or friends□ Professional literature

□ Others

□ Never heard of it

21. Have you learned about the protective effect of COVID-19 inhaled vaccine recently? [Single choice]

○ Fully understanding

○ Well understanding

○ Understand

○ Heard of, but don't understand

○ Never heard of it at all

22. Have you recently learned about the protective effect of COVID-19 nasal spray vaccine? [Single choice]

○ Fully understanding

○ Well understanding

○ Understand

○ Heard of, but don't understand

○ Never heard of it at all

23. In your opinion, the main advantages of COVID-19 nasal spray vaccine and inhalation vaccine over intramuscular injection vaccine lie in [up to three items] [multiple choice]

□ No injection /pain

□ Long duration of immune response

□ Can induce mucosal immunity

□ More effective blocking of COVID-19 infection

□ Self-service vaccination and save resources

□ Less side effects

□ Reduce the use of needles and medical waste

□ Others, please specify_________________

□ Not sure/I don’t know

Depends on item 1 of question 21; 2; 3; 4 options, item 1 of question 22; 2; 3; 4 options*

24. In your opinion, the main disadvantages of COVID-19 nasal spray vaccine and inhalation vaccine compared with intramuscular vaccine lie in [up to three items] [multiple choice]

□ Slow perform

□ Vulnerable to external influences (cough, sneeze, etc.)

□ The mechanism of action is not completely clear, and safety is unknown

□ Not-easy-control dose (incomplete spray/inhalation, incomplete atomization, etc.)

□ Generate new medical waste

□ Contraindications may need to be considered more

□ Inoculation operation is not convenient

□ Others, please specify_________________

□ Uncertain/unclear

Depends on item 1 of question 21; 2; 3; 4 options, item 1 of question 22; 2; 3; 4 options*

25. If there are multiple vaccination methods for you to choose at will, what are the main factors you will consider? [Up to three items] [multiple choice]

□ Safety

□ Vaccine efficacy

□ Easy to gain and vaccination

□ price

□ Understanding level

□ Painless vaccination

□ National recommendations

□ Suggestions from friends or doctors

□ Other factors, please specify_________________

26. If the country recommends the booster dose of COVID-19 vaccine (the third dose, the fourth dose, etc.), which immunization routes would you like to vaccinate? [Single choice]

○ It doesn't matter, use whatever I have

○ intramuscular injection

○ Non-intramuscular injection method (such as nasal spray or inhalation)

○ Neither is willing, but hopes to have other better vaccination methods

○ Have not been vaccinated with COVID-19 vaccine

○ Not willing to receive more doses of COVID-19 vaccine

China has covered two doses of COVID-19 vaccination, and recommended the third booster dose. In the future, COVID-19 vaccine booster doses may have various immunization routes, including intramuscular injection, nasal spray or inhalation. Please read the following question and choose the most suitable option. Each question has five options, from left to right: 1. Strongly disagree 2. Disagree 3. Neutral/don't know 4. Agree 5. Strongly agree

27. Receive COVID-19 booster can protect my health. [single choice]

Strongly disagree ○ 1 ○ 2 ○ 3 ○ 4 ○ 5 Strongly agree

28. The doses of COVID-19 vaccine I have received is enough, and I don’t need to receive another booster dose. [single choice]

Strongly disagree ○ 5 ○ 4 ○ 3 ○ 2 ○ 1 Strongly agree

29. The COVID-19 pandemic in China has been control, so there is no need for booster vaccination [single choice]

Strongly disagree ○ 5 ○ 4 ○ 3 ○ 2 ○ 1 Strongly agree

30. Please choose "4" [single choice]

Strongly disagree ○ 1 ○ 2 ○ 3 ○ 4 ○ 5 Strongly agree

31. I think that accepting COVID-19 vaccine booster have strongly effective on preventing COVID-19 [single choice]

Strongly disagree ○ 1 ○ 2 ○ 3 ○ 4 ○ 5 Strongly agree

32. Inoculation of COVID-19 vaccine booster can protect people around me (family, friends, colleagues) from infection [single choice]

Strongly disagree ○ 1 ○ 2 ○ 3 ○ 4 ○ 5 Strongly agree

33. If there is a booster vaccination for the new SARS-CoV-2 variants, I will immediately try to obtain [single choice]

Strongly disagree ○ 1 ○ 2 ○ 3 ○ 4 ○ 5 Strongly agree

34. I am still worried about the possible side effects after the vaccination of booster [single choice]

Strongly disagree ○ 5 ○ 4 ○ 3 ○ 2 ○ 1 Strongly agree

35. If the government, doctors, community and other professionals recommend the vaccination of booster, I believe that vaccination is beneficial [single choice]

Strongly disagree ○ 1 ○ 2 ○ 3 ○ 4 ○ 5 Strongly agree

36. I will recommend people around me (family, friends, colleagues) to actively vaccinate each dose of COVID-19 vaccine [single choice]

Strongly disagree ○ 1 ○ 2 ○ 3 ○ 4 ○ 5 Strongly agree

37. If I need to be self-funded or partially self-funded to vaccinate the booster, I may not choose to vaccinate [single choice]

Strongly disagree ○ 5 ○ 4 ○ 3 ○ 2 ○ 1 Strongly agree
